# Frequency and socio-demographic correlates of eating meals out and take-away meals at home: cross-sectional analysis of the UK national diet and nutrition survey, waves 1–4 (2008–12)

**DOI:** 10.1186/s12966-015-0210-8

**Published:** 2015-04-16

**Authors:** Jean Adams, Louis Goffe, Tamara Brown, Amelia A Lake, Carolyn Summerbell, Martin White, Wendy Wrieden, Ashley J Adamson

**Affiliations:** Centre for Diet & Activity Research, MRC Epidemiology Unit, University of Cambridge, School of Clinical Medicine, Box 285 Institute of Metabolic Science, Cambridge Biomedical Campus, Cambridge, CB2 0QQ UK; Institute of Health & Society, Newcastle University, Baddiley-Clark Building, Richardson Road, Newcastle upon Tyne, NE2 4AX UK; Human Nutrition Research Centre, Newcastle University, William Leech Building, Claremont Place, Newcastle upon Tyne, NE2 4AH UK; Wolfson Research Institute, Durham University, Queen’s Campus, University Boulevard, Stockton on Tees, Thornaby, TS17 6BH UK

**Keywords:** Diet, Eating out, Food, Meals, Restaurant, Socioeconomic, Take-away

## Abstract

**Background:**

Food prepared out-of-home tends to be less healthful than food prepared at home, with a positive association between frequency of consumption and both fat intake and body fatness. There is little current data on who eats out-of-home food. We explored frequency and socio-demographic correlates of eating meals out and take-away meals at home, using data from a large, UK, population representative study.

**Methods:**

Data were from waves 1–4 of the UK National Diet and Nutrition Survey (2008–12). Socio-demographic variables of interest were gender, age group, and socio-economic position. Self-reported frequency of consuming meals out and take-away meals at home was categorised as: less than once per week and once per week or more. Analyses were performed separately for adults (aged 18 years or older) and children.

**Results:**

Data from 2001 adults and 1963 children were included. More than one quarter (27.1%) of adults and one fifth (19.0%) of children ate meals out once per week or more. One fifth of adults (21.1%) and children (21.0%) ate take-away meals at home once per week or more. There were no gender differences in consumption of meals out, but more boys than girls ate take-away meals at home at least weekly. The proportion of participants eating both meals out and take-away meals at home at least weekly peaked in young adults aged 19–29 years. Adults living in more affluent households were more likely to eat meals out at least once per week, but children living in less affluent households were more likely to eat take-away meals at home at least once per week. There was no relationship between socio-economic position and consumption of take-away meals at home in adults.

**Conclusions:**

One-fifth to one-quarter of individuals eat meals prepared out-of-home weekly. Interventions seeking to improve dietary intake by reducing consumption of out-of-home food may be more effective if tailored to and targeted at adults aged less than 30 years. It may also be important to develop interventions to help children and adolescents avoid becoming frequent consumers of out-of-home food.

## Background

Out-of-home sources of ready-to-eat food include vending machines, take-aways, cafes, restaurants, supermarkets and convenience stores [1]. Food prepared out-of-home tends to be less healthful than food prepared at home, particularly in terms of energy and fat content [2]. This likely explains the associations found between frequency of eating out-of-home food and both nutritional quality of total diet and body weight [2,3].

Eating food prepared out-of-home is becoming more common across the world and makes a substantial contribution to individual diets and household spending on food [2,4-6]. For example, in 2012, about 10% of total daily energy intake of UK individuals was accounted for by food prepared and consumed out-of-home, with up to an additional 4% accounted for by take-away food eaten at home [7]. Between 2009 and 2012, expenditure on take-away food eaten at home rose by 11%, in real terms, to an average of £1.79 ($US2.86, €2.28) per person per week [7]. Over the same period, spending on eating out (excluding alcoholic drinks) rose by 7% to £8.95 ($US14.29, €11.40) per person per week, accounting for more than one quarter (26%) of total household spending on food and non-alcoholic drinks [7].

In terms of socio-demographic correlates of out-of-home food consumption, the scant, available data from developed countries suggests that men tend to consume more of their total dietary energy away from home [8,9], and obtain more meals from out-of-home sources [5] than women. Consumption of out-of-home food tends to increase with age in children, peak in late adolescence or early adulthood [10], and then drop with increasing age in adulthood [5,8,9,11-13]. An inconsistent relationship between markers of socio-economic position and out-of-home food consumption in developed countries was found in a systematic review [2].

Interventions to improve the nutritional content of food prepared out-of-home have been attempted in the UK and elsewhere [14,15]. Governmental public health and health education agencies are also increasingly urging individuals to cut down on their consumption of food prepared out-of-home, particularly fast food [16]. As reviewed above, some evidence of differences in consumption of out-of-home food according to socio-demographic characteristics [17,18] is emerging. However, little data from the UK exists. Better understanding of socio-demographic patterns of out-of-home eating is needed to more effectively target and tailor interventions.

Two key problems exist within the literature on out-of-home eating. First, variations in the definition of out-of-home eating make comparisons between studies difficult [2,4]. Some authors focus on food consumed out-of-home, irrespective of where it was prepared; (e.g. [8-10,12]) whilst others focus on food prepared out-of-home, irrespective of where it was consumed (e.g. [5,11,13,19]). Second, international differences in dietary intake [20] and out-of-home eating patterns [8] mean data from one country may not be generalisable to others.

To add to the sparse literature on who eats out-of-home food, we explored frequency and socio-demographic correlates of eating meals out and take-away meals at home, using data from a large, UK, population-representative study. We particularly focused on food prepared, rather than consumed, out-of-home as food prepared out-of-home is the subject of current policy focus [16].

## Methods

We performed a cross-sectional analysis of the first four years of data from the UK National Diet and Nutrition Survey (NDNS).

### Data source and participants

The NDNS is an annual, cross sectional survey collecting information on the food consumption, nutrient intakes and nutritional status of people aged 1.5 years and older living in private households in the UK. The current programme began in 2008–09 and recruits around 1000 participants per year – 500 children aged 1.5-18 years, and 500 adults aged 19 years or older. As far as possible, sampling, recruitment and data collection methods stay constant from year to year allowing data to be combined across survey years.

Households across the UK are selected to take part in the NDNS using a multi-stage probability design. In each wave, a random sample of primary sampling units is selected for inclusion. These primary sampling units are small geographical areas that allow more efficient data collection by enabling it to be geographically focused. Within these primary sampling units, private addresses are randomly selected for inclusion. If, on visiting, it is found that more than one household lives at a particular address, one is randomly selected for inclusion. Within participating households, up to one adult and one child are randomly selected to take part. Data collection involves a researcher interview covering socio-demographics and shopping, cooking and eating habits; participant completion of a four-day food diary; and a nurse visit. Parents, or carers, provide information on children aged 11 years and younger [21].

Overall, 91% of households eligible for inclusion agreed to take part in the first four waves of NDNS. Usable food diaries (three or four completed days) were collected from at least one household member in 58% of eligible households. At an individual level, 56% of those selected to take part completed usable food diaries: 2083 adults and 2073 children [21].

Anonymised data from the first four waves (2008–09 to 2011–12) of the NDNS were obtained from the UK Data Archive – a data sharing service for the UK research community. These data are available to other eligible researchers directly from the Archive.

### Variables of interest

#### Frequency of eating meals out and take-away meals at home

Whilst the NDNS food diary includes information on where food was consumed, it does not collect information on where food was obtained. However, the researcher interview includes two questions on frequency of eating food prepared out-of-home. These are: “On average, how often do you/does child eat meals out in a restaurant or cafe?”; and “On average, how often do you/does child eat take-away meals at home?”. In both questions it is specified that “‘meals’ means more than a beverage or bag of chips” and participants are asked to “include pizza, fish and chips, Indian, Chinese, burgers, kebab etc.”. Response options are: “rarely or never”, “1-2 times per month”, “1-2 times per week”, “3-4 times per week”, and “5 or more times per week”. We believe these questions were designed specifically for the NDNS. Information is not available on the reliability or validity of these questions, but similar frequency-based questions have been used previously [5].

Responses in the two least frequent categories of the above questions (rarely or never, and 1–2 times per month) were collapsed for analysis as previous research indicates that the health risks associated with fast food occur in those with frequent consumption (more than once per week) [22]. As 5% or fewer participants consumed meals out or take-away meals at home more than 1–2 times per week, responses in the three most frequent categories (1–2 times per week, 3–4 times per week, and 5 or more times per week) were also combined for analysis. This led to dichotomous outcome variables with categories of: less than once per week; and once per week or more.

#### Socio-demographic variables

Three socio-demographic variables were included: age in years, gender, and socio-economic position. Age was collapsed into approximately 5-year categories for children, and 10-year categories for adults.

Socio-economic position (SEP) was measured using household occupational social class and, in adults only, individual age at completion of full time education. Although net household income (another common measure of SEP) [17,18] is collected in NDNS, around 15% of participants refuse the question and this level of attrition was not considered acceptable.

Occupational social class was categorised using the three-level National Statistics Socio-economic Classification [23] of the household as: higher managerial, administrative and professional occupations; intermediate occupations; and routine and manual occupations. This classification assigns all individuals living in the same household according to the occupation of the householder - the individual responsible for owning or renting the accommodation. Where there is more than one householder, the individual with the highest income takes precedence. Where more than one householder has the same income, the oldest individual takes precedence. Unemployed householders are categorised according to their last main occupation. A small number of households are excluded from this classification where the householder has never worked.

Individual age at completion of full time education was categorised as less than 16 years (equivalent to having obtained less than basic school leaving qualifications), 16–17 (equivalent to basic school leaving qualifications), or more than 18 years (equivalent to advanced school leaving qualifications or more). This variable was not calculated for children aged 18 years or younger as many have not completed full time education. Education was used as an individual, rather than household, marker of SEP. As such we did not use parental education as a proxy for children as it would not be clear which parents’ educational status to apply.

### Data analysis

Individuals were included in the analysis if they took part in the NDNS in waves 1–4 and information on all of the variables of interest was available.

Logistic regression was used to explore the unadjusted, and mutually adjusted, relationships between likelihood of eating meals out or take-away meals at home once per week or more often across socio-demographic variables. As there was evidence that different patterns in proportion of participants eating these meals once per week or more according to age in adults and children, all analyses were conducted separately for children (18 years or younger) and adults (older than 18 years).

Study weights, prepared by NDNS and provided with the data, were used to account for selective non-response. These weights take account of the fact that whilst sampling for the NDNS is population-representative, response is not. Some population groups are less likely to respond to the invitation to take part in the NDNS than others. Applying study weights corrects for this selective non-response and returns the results to population-representative. The use of study weights mean that percentages (with 95% confidence intervals) are presented rather than raw frequencies.

All analyses were conducted in Stata v13.

### Ethics

Ethical approval for collection of NDNS data was provided by Oxfordshire A Research Ethics Committee. Ethical approval for this secondary analyses of anonymised data was not required.

## Results

Of 2083 adults and 2073 children who took part in the NDNS in waves 1–4, 2001 (96.1%) adults and 1963 (94.7%) children were included in the analyses. The distribution of participants across socio-demographic variables is shown in the first data column of Table 1.

**Table 1:** **Frequency of eating meals out by socio-demographic variables; UK National Diet & Nutrition Survey 2008-12**

**Variable**	**Level**	**Distribution, %** ^**1**^ **(95% CI)**	**1-2/week or more, % (95% CI)**	**Unadjusted OR (95% CI)**	**Mutually adjusted OR (95% CI)**
**All adults (n = 2001)**		100	27.1 (25.0 – 29.4)	--	--
**Gender**	Male	49.1 (46.7 – 51.6)	28.8 (25.5 – 32.2)	Reference	Reference
	Female	50.9 (48.4 – 53.3)	25.6 (22.8 – 28.6)	0.90 (0.70 – 1.15)	0.89 (0.70 – 1.14)
**Age (years)**	19-29	18.2 (16.1 – 20.5)	41.0 (34.4 – 47.9)	Reference	Reference
	30-39	17.4 (15.7 – 19.2)	21.9 (17.7 – 26.7)	**0.39 (0.26 – 0.58)**	**0.35 (0.23 – 0.54)**
	40-49	19.2 (17.4 – 21.1)	24.7 (20.5 – 29.4)	**0.47 (0.32 – 0.70)**	**0.43 (0.29 – 0.65)**
	50-59	15.8 (14.2 – 17.6)	25.7 (21.1 – 31.0)	**0.51 (0.34 – 0.77)**	**0.48 (0.32 – 0.73)**
	60-69	14.0 (12.4 – 15.7)	20.1 (15.3 – 25.8)	**0.36 (0.22 – 0.57)**	**0.34 (0.21 – 0.56)**
	>69	15.4 (13.7 – 17.3)	27.6 (22.3 – 33.7)	**0.58 (0.37 – 0.90)**	**0.57 (0.35 – 0.90)**
**NS-SEC**	Routine & manual	35.4 (33.1 – 37.8)	20.8 (17.7 – 24.3)	Reference	Reference
	Intermediate	20.5 (18.6 – 22.5)	29.7 (24.8 – 35.0)	**1.76 (1.24 – 2.49)**	**1.89 (1.33 – 2.69)**
	Managerial & professional	44.1 (41.7 – 46.6)	31.1 (27.7 – 34.7)	**1.81 (1.37 – 2.41)**	**1.95 (1.43 – 2.65)**
**Age left education (years)**	<16	24.1 (21.9 – 26.5)	24.2 (19.8 – 29.3)	Reference	Reference
	16-17	35.5 (33.0 – 38.0)	25.7 (22.0 – 29.7)	1.08 (0.78 – 1.50)	0.97 (0.68 – 1.40)
	>17	40.4 (37.8 – 43.0)	29.5 (25.7 – 33.6)	1.31 (0.95 – 1.80)	1.01 (0.69 – 1.48)
**All children (n = 1963)**		100	19.0 (17.2 – 21.0)	--	--
**Gender**	Male	52.0 (49.5 – 54.4)	20.0 (17.4 – 22.8)	Reference	Reference
	Female	48.0 (45.6 – 50.5)	18.0 (15.5 – 20.8)	0.88 (0.67 – 1.14)	0.87 (0.66 – 1.13)
**Age (years)**	0-4	19.6 (17.9 – 21.5)	21.0 (17.4 – 25.1)	Comparator	Comparator
	5-9	28.4 (26.2 – 30.6)	17.3 (14.2 – 21.0)	0.82 (0.57 – 1.16)	0.81 (0.57 – 1.16)
	10-14	28.4 (26.2 – 30.8)	13.5 (10.6 – 17.0)	**0.61 (0.42 – 0.89)**	**0.61 (0.42 – 0.89)**
	15-18	23.5 (21.4 – 25.8)	26.1 (21.8 – 31.0)	1.25 (0.87 – 1.81)	1.27 (0.88 – 1.82)
**NS-SEC**	Routine & manual	37.0 (34.6 – 39.5)	17.9 (15.0 – 21.3)	Reference	Reference
	Intermediate	20.0 (18.1 – 22.1)	21.4 (17.3 – 26.2)	1.34 (0.93 – 1.94)	1.35 (0.93 – 1.96)
	Managerial & professional	43.0 (40.6 – 45.4)	18.8 (16.2 – 21.7)	1.10 (0.81 – 1.48)	1.12 (0.83 – 1.52)

^1^weighted percentages that correct for selective non-response.

CI: confidence intervals; NS-SEC: National Statistics Socio-economic Classification.

Bold indicates statistically significantly different from reference category at p < 0.05.

The proportion of participants who ate meals out once per week or more, overall and by socio-demographic variables, is shown in Table 1. Just over one-quarter of adults (27.1%) and just less than one-fifth of children (19.0%) ate meals out once per week or more. There were no gender differences in proportions eating meals out once per week or more in adults or children. In adults, eating meals out once per week or more was most common in the youngest age groups (19–29 years), with significantly more participants in this group (41.0%) eating meals out once per week or more than in other groups (20.1%-27.6%) in unadjusted and mutually adjusted analyses. However, there was no evidence of an inverse linear association with age. Significantly fewer children aged 10–14 years ate meals out once per week or more than those in the youngest age group (1.5 – 4 years), but there were no other statistically significant differences by age in children.

Adults living in households in the intermediate and most affluent occupational social class were significantly more likely to eat meals out once per week or more than those in the lowest social class in both unadjusted and adjusted analyses. However, there was no difference in the proportion of adults eating meals out once per week or more by individual education in adults, or by household occupational social class in children.

Table 2 shows the proportion of participants who ate take-away meals at home once per week or more. More than one fifth of adults (21.1%) and children (21.0%) ate take-away meals at home this frequently.

**Table 2:** **frequency of eating take away meals at home by socio-demographic variables; UK National Diet & Nutrition Survey 2008-12**

**Variable**	**Level**	**1-2/week or more, % (95% CI)**	**Unadjusted OR (95% CI)**	**Mutually adjusted OR (95% CI)**
**All adults (n = 2001)**		21.1 (19.1 – 23.3)	--	--
**Gender**	Male	23.1 (20.1 – 26.4)	Reference	Reference
	Female	19.2 (16.6 – 22.0)	0.89 (0.68 – 1.17)	0.94 (0.71 – 1.23)
**Age (years)**	19-29	32.4 (26.3 – 39.1)	Reference	Reference
	30-39	25.8 (21.3 – 30.9)	0.82 (0.54 – 1.26)	0.81 (0.53 – 1.23)
	40-49	26.5 (22.1 – 31.4)	0.96 (0.64 – 1.45)	0.90 (0.59 – 1.36)
	50-59	16.9 (12.9 – 21.8)	**0.50 (0.32 – 0.80)**	**0.49 (0.31 – 0.78)**
	60-69	12.6 (8.6 – 17.9)	**0.38 (0.22 – 0.66)**	**0.36 (0.21 – 0.64)**
	>69	7.9 (4.7 – 12.9)	**0.19 (0.09 – 0.39)**	**0.18 (0.08 – 0.37)**
**NS-SEC**	Routine & manual	22.1 (18.8 – 25.8)	Reference	Reference
	Intermediate	23.5 (19.0 – 28.8)	1.03 (0.71 – 1.51)	1.09 (0.73 – 1.60)
	Managerial & professional	19.2 (16.4 – 22.3)	0.99 (0.73 – 1.34)	1.03 (0.74 – 1.44)
**Age left education (years)**	<16	14.5 (10.9 – 19.0)	Comparator	Comparator
	16-17	25.8 (22.0 – 29.9)	**2.04 (1.39 – 3.00)**	1.25 (0.83 – 1.89)
	>17	19.9 (16.8 – 23.5)	1.47 (0.99 – 2.17)	0.83 (0.53 – 1.31)
**All children (n = 1963)**		21.0 (19.1 – 23.1)	--	--
**Gender**	Male	23.7 (20.8 – 26.7)	Reference	Reference
	Female	18.2 (15.6 – 21.1)	**0.71 (0.55 – 0.93)**	**0.70 (0.54 – 0.91)**
**Age (years)**	0-4	14.0 (10.8 – 17.9)	Reference	Reference
	5-9	18.4 (15.1 – 22.1)	1.43 (0.96 – 2.11)	1.44 (0.98 – 2.14)
	10-14	23.1 (19.3 – 27.4)	**1.77 (1.20 – 2.61)**	**1.79 (1.20 – 2.64)**
	15-18	27.7 (23.1 – 32.7)	**2.43 (1.63 – 3.64)**	**2.44 (1.63 – 3.65)**
**NS-SEC**	Routine & manual	26.0 (22.6 – 29.9)	Reference	Reference
	Intermediate	20.4 (16.1 – 25.4)	0.81 (0.56 – 1.17)	0.82 (0.57 – 1.19)
	Managerial & professional	17.0 (14.4 – 19.9)	**0.59 (0.44 – 0.79)**	**0.60 (0.45 – 0.81)**

^1^weighted percentages that correct for selective non-response.

OR: odds ratio; CI: confidence intervals; NS-SEC: National Statistics Socio-economic Classification.

Bold indicates statistically significantly different from reference category at p < 0.05.

There were no gender differences in the proportion of participants who ate take-away meals at home once per week or more in adults, but girls were less likely to eat these meals frequently than boys – in unadjusted and mutually adjusted analyses. In adults, the proportion of participants eating take-away meals at home once per week or more tended to decrease with age. Adults in the oldest group (more than 69 years) were one fifth as likely to eat these meals once per week or more than those aged 19–29 years. In children, the proportion eating take-away meals at home once per week or more increased with age. Children aged older than 9 years were significantly more likely to eat these meals at least once per week than those aged less than 5 years in both unadjusted and mutually adjusted analyses. Children aged 15–18 years were more than twice as likely to eat these meals once per week or more than children aged 1.5 – 4 years.

There was no difference in the proportion of adults eating take-away meals at home once per week or more by household occupation social class or individual education in adults after mutual adjustment. However, children living in households of the highest occupational social class were significantly less likely to eat take-away meals at home once per week or more than those living in the least affluent households.

Table 3 shows cross-tabulations of eating meals out and take-away meals at home in adults and children. There was evidence that both adults and children who ate meals out at least once per week were also more likely to eat take-away meals at home at least once per week.

**Table 3:** **Cross-tabulation of frequency of eating meals out and take-away meals at home; UK National Diet & Nutrition Survey 2008-12**

**Meals out**	**Take-away meals at home**			**Chi-squared (df), p-value**
	**<1/week**	**1-2/week or more**	**Total**	
**All adults (n = 2001)**	78.9	21.1	100	
**<1/week**	60.0	12.9	72.9	
**1-2/week or more**	18.9	8.2	27.1	18.39 (1), <0.001
**All children (n = 1963)**	79.0	21.0	100	
**<1/week**	66.9	14.1	81.0	
**1-2/week or more**	12.1	6.9	19.0	45.66 (1), <0.001

Figures show % of all participants in each cell, weighted for selective non-response.

## Discussion

### Statement of principal findings

As far as we are aware, this is the first description of frequency and socio-demographic correlates of eating food from out-of-home sources in a UK population-representative cohort. In terms of frequency of consumption, around one quarter of adults and one fifth of children ate meals out once per week or more; around one fifth of adults and children ate take-away meals at home once per week or more. In terms of socio-demographic correlates, the only gender differences were that boys were more likely to eat take-away meals at home at least once per week than girls. The proportion of participants who ate both meals out and take-away meals at home at least once per week peaked at age 19–29 years and decreased in older adults. Adults living in households of higher occupational social class were more likely to eat meals out at least once per week, but there was no difference by individual education in adults or household social class in children. Noticeably, there were no socio-economic differences in the proportion of adults eating take-away meals at home at least once per week. But, children living in households of the highest occupation social class were less likely to eat take-away meals at home at least once per week than those in the lowest.

### Interpretation and implications of findings

In a number of countries, governments provide advice on how to choose more healthful options when eating meals out and choosing take-away meals, but no guidance on the maximum recommended frequency of consumption is provided [24,25]. Studies have found that the health risks associated with fast food occur in those with consumption more than once per week [22]. If ‘fast food’ and ‘take-away’ food can be equated, our findings indicate this level of consumption is particularly common in certain population sub-groups. In particular, almost one-third of adults aged 19–29 years ate take-away meals at home this often. Consumers, particularly aged 19–29 years, may benefit from interventions to decrease frequency of consumption.

Differences in methods also make it hard to compare frequency of consumption reported here with previous work. The most comparable previous data reports on frequency of eating meals prepared in restaurants (either sit-in or take-away) amongst adults in the USA – where 41% of US adults ate such meals three times or more per week in 2000 [5]. As 5% or less of participants in the current study consumed meals out or take-away meals at home more than 1–2 times per week, this suggests that consumption is substantially higher in the USA than the UK. Further consideration of why individuals consume meals out and take-away meals at home, and if this differs between the USA and UK, could inform the development of interventions to prevent further increases in, and even reduce, consumption in both settings, and elsewhere.

Changes in consumption of meals out and take-away meals at home over time may also limit comparisons with previous studies. There is evidence that the prevalence of out-of-home eating establishments increased between 1980 and 2000 [26], suggesting that consumption has also increased over recent decades.

Our finding that boys were more likely than girls to eat take-away meals at home, but not meals out, frequently reflects previous findings. Others have reported that men obtain a greater proportion of their total energy from food consumed out-of-home [8,9], and consume commercially prepared foods more often [5]. The distinctions between meals out and take-away meals at home, and gender differences in children, have not been previously studied. Unlike previous work [5,8,9], we did not find the same gender difference in frequency of consumption of either type of meal in adults. It is possible that patterns of out-of-home eating by gender may have changed in recent years.

Our finding that consumption of both meals out and take-away meals at home peak in early adulthood (19–29 years) is similar to previous reports [5,8,9,11-13]. However, few previous studies have explored the relationship with age in children, and only one has included both adults and children [11]. These trends may reflect true changes in consumption over the lifecourse, secular trends in consumption, or a mixture of both. For example, older people may be less likely to eat meals from out-of-home sources because they tend to have less disposable income than others [27]; because they did not develop the habit earlier in their life when such options were less common [5]; or for a combination of these, and other, reasons.

We did not study the nutritional quality of the out-of-home meals consumed by participants. It would be inappropriate to assume that all meals from out-of-home sources are nutritionally equal and there may be systematic differences in what is consumed by socio-demographic characteristics. Interventions seeking to improve dietary intake by reducing consumption of out-of-home food may be more effective if tailored to, and perhaps targeting at, adults aged less than 30 years. It may also be important to develop interventions to help children and adolescents avoid becoming frequent out-of-home food consumers.

Eating meals out at least once per week was particularly high in the youngest children (1.5-4 years) (21.0%). This may reflect that these children spend little or no time in formal educational settings meaning they have more opportunity than others to accompany their parents to lunches out, in particular.

Adults of higher SEP, measured by household occupational class, but not individual education, were most likely to eat meals out at least once per week. This may reflect the differences in what different measures of SEP capture. Household occupational social class is more often a measure of current status than educational attainment – which may reflect circumstances many years in the past. [17,18] Differences in eating meals out by occupational class may reflect that such meals can be expensive (although comparative data on the cost of meals out, take-away meals, and home cooked meals is not readily available), as well as cultural differences in leisure activities [28].

No differences in consumption of take-away meals at home were seen in adults according to either measure of SEP. However, children living in households of higher occupational class were less likely to eat take-away meals once per week or more than those living in households of the lowest occupational class. This is perhaps contrary to popular beliefs where high take-away consumption in less affluent groups (across the board, and not just in children) is suggested to contribute to socio-economic inequalities in obesity [29,30]. As noted, previous studies have reported inconsistent associations between SEP and out-of-home eating in adults [2]. Our results suggest this may be due to differences in definition of out-of-home eating, with different relationships seen for meals out and take-away meals at home; or differences in operationalization of SEP, with different relationships seen for occupational social class and education (in adults). It is possible that socio-economic trends in take-away consumption in today’s children will carry forward to tomorrow’s adults.

We studied meals out and take-away meals separately as there is reason to believe these represent different types of eating [28,31]. Whilst the same food can be bought to take-away or eat in-store in some outlets, many outlets will provide only food to take-away or only food to eat in [1]. Furthermore, the prevalence of these different types of outlets varies with socio-economic markers of local areas. In particularly, restaurants for consuming meals out are most common in more affluent areas in the UK [32].

The fact eating take-away meals at home was more common in less affluent children, but not adults, suggests that socio-economic influences on consumption are not consistent across the lifecourse. Take-away outlet density is higher in more deprived UK neighbourhoods [33], and everyday exposure to take-away outlets across home and work neighbourhoods and commuting routes has been associated with consumption in UK adults [34]. It is possible that children are less mobile in their daily lives and so are more exposed to their home neighbourhood environment than adults. Thus, children who live in deprived neighbourhoods may be particularly exposed to the take-away outlets concentrated there, whereas more mobile adults living in the same neighbourhoods may have more variable take-away outlet exposure as they spend time at home, at work, and commuting.

### Strengths and limitations of methods

The NDNS aims to recruit a population-representative sample at each wave and study weights are provided to take account of selective non-response where this exists. Our results are, therefore, likely to be generalisable across the UK. However, given known international differences in eating patterns [8,20], they may not be generalisable to other contexts. Further work, using consistent methods, is required to provide internationally comparable information. The large sample size of both children and adults means that our analyses are unlikely to be underpowered.

The current data were collected in 2008–12, making this the most up-to-date UK dataset on this topic. Whilst it is possible that more recent data may reveal different trends, there was no evidence of differences in frequency of consumption over the four survey years included (data not shown).

Although NDNS does sometimes include one adult and one child per household and it would be possible, in some cases, to explore household congruence of out-of-home eating patterns, this was out with the scope of the current work. Future work could explore household, and other influences, on eating out-of-home.

The validity and reliability of the questions used to assess frequency of consumption have not been explored. One previous study found that frequency of eating out-of-home food in children aged 5–12 years tended to be under-reported in questionnaire compared to food-diary data [10]. Any systematic differences in interpretations across socio-demographic groups would lead to bias, but the nature of this is difficult to predict. Furthermore, the questions used do not capture the consumption of all food prepared out-of-home. Ideally, the source of all food consumed would be captured alongside food diary data. This could be included in future waves of NDNS.

Out-of-home food outlets are heterogeneous. No information was collected on the specific type of meal out or take-away outlets visited and there may be systematic differences in these across population groups. Similarly, although guidance was given on what a ‘meal’ is, differences in individual interpretations are likely to introduce error. Future work that disaggregates out-of-home eating using existing tools [32] may reveal further, interesting patterns.

We measured SEP using household occupational social class and, in adults only, age at leaving full time education. These capture two of the three most common measures of individual SEP – occupation, education and income. We were unable to use a marker of individual educational attainment in children as the great majority were still in full time education. Although household income is captured in NDNS, around 15% of the sample refused this particular question – leading to potential non-response bias. We did not include a measure of income for this reason.

It was beyond the scope of the current work to explore the relationships between frequency of eating meals out and take-away meal at home and either dietary quality or adiposity. This has been explored in other (non-UK) cohorts [3,22] and future work could explore these relationships further within NDNS.

## Conclusions

We studied frequency and socio-demographic correlates of eating meals out and take-away meals at home, using data from a large, UK population-representative study. Between a fifth and a quarter of people in the UK eat meals out once per week or more, with one fifth eating take-away meals at home this frequently. Boys were more likely than girls to eat take-away meals at home frequently, but no other gender differences were found. At least weekly consumption of both types of meal peaks in early adulthood. Adults from more affluent households are more likely to eat meals out at least weekly, but children from less affluent households are more likely to eat take-away meals at home at least weekly. Interventions seeking to improve dietary intake by reducing consumption of out-of-home food may be more effective if tailored to, or targeting at, adults aged less than 30 years. It may also be important to develop interventions to help children and adolescents avoid becoming frequent consumers of out-of-home food.
